# Adaptation and validation of a German version of the Multimorbidity Treatment Burden Questionnaire

**DOI:** 10.1186/s12955-022-01993-z

**Published:** 2022-06-03

**Authors:** Josefine Schulze, Amanda Breckner, Polly Duncan, Martin Scherer, Nadine Janis Pohontsch, Dagmar Lühmann

**Affiliations:** 1 Department of General Practice and Primary Care, University Medical Center Hamburg-Eppendorf, Martinistraße 52, Hamburg, 20246 Germany; 2 Centre for Academic Primary Care, University of Bristol, Bristol, UK; 3 Department of General Practice and Health Services Research, Heidelberg University Hospital, Heidelberg, Germany

**Keywords:** Primary care, Comorbidity, Quality of life, Patient-reported outcome, Patient-centred care, Adherence

## Abstract

**Background:**

Patients with multiple long-term conditions often face a variety of challenges arising from the requirements of their health care. Knowledge of perceived treatment burden is crucial for optimizing treatment. In this study, we aimed to create a German version of the Multimorbidity Treatment Burden Questionnaire (MTBQ) and to evaluate its validity.

**Methods:**

The steps to translate the MTBQ included forward/back translation, cognitive interviews (*n* = 6) and a pilot test (*n* = 7). Psychometric properties of the scale were assessed in a cross-sectional survey with primary care patients aged 65 and older with at least 3 long-term conditions (*n* = 344). We examined the distribution of responses, dimensionality, internal reliability and construct validity.

**Results:**

Cognitive interviewing and piloting led to minor modifications and showed overall good face validity and acceptability. As expected, we observed a positively skewed response distribution for all items. Reliability was acceptable with McDonald’s omega = 0.71. Factor analysis suggested one common factor while model fit indices were inconclusive. Predefined hypotheses regarding the construct validity were supported by negative associations between treatment burden and health-related quality of life, self-rated health, social support, patient activation and medication adherence, and positive associations between treatment burden and number of comorbidities. Treatment burden was found to be higher in female participants (*Mdn*_1_ = 6.82, *Mdn*_2_ = 4.55; *U* = 11,729, *p* = 0.001) and participants with mental health diagnoses (*Mdn*_1_ = 9.10, *Mdn*_2_ = 4.55; *U* = 3172, *p* = 0.024).

**Conclusions:**

The German MTBQ exhibited good psychometric properties and can be used to assess the perceived treatment burden of patients with multimorbidity.

**Supplementary Information:**

The online version contains supplementary material available at 10.1186/s12955-022-01993-z.

## Background

More and more elderly people are affected by multiple health problems and face a significant burden in managing these conditions [1, 2]. Patients with multimorbidity are often seen by multiple primary and secondary healthcare professionals, each advising different treatment, including long-term medications and recommendations for diet and exercise. There is a growing body of literature recognizing the critical role of treatment burden in chronic care [3]. This concept describes the workload of health care-related tasks and its impact on patients’ daily lives and their well-being [4]. There is a positive correlation between treatment burden and the number of conditions [5, 6], especially in discordant conditions with different pathophysiologic profiles and/or treatment strategies. Tran et al. [7] found that about 40% of people with long-term conditions do not feel able to sustain the present efforts for their care in the future. Although patients with a poor overall health status spend more than 3.5 h per week on health-related self-care, clinicians tend to overlook their workload [8].

Boyd et al. [9] demonstrated in their classic example of a hypothetical 79-year-old woman with diabetes, chronic obstructive pulmonary disease (COPD), osteoporosis, hypertension and osteoarthritis how following single clinical practice guidelines can lead to a complex and contradictory treatment regimen with 19 daily doses of medication and 14 non-pharmacological recommendations. The authors concluded that for patients with complex care needs, individual treatment burden, potential risks and benefits should be taken into account in a shared decision-making process. Shippee et al. [10] used the *Cumulative Complexity Model* to illustrate that overburdening and non-adherence occur when patients’ treatment-related workload is greater than their capacity to cope with these demands. A recent systematic review outlines that treatment burden is determined not only by health care-related workload and individual abilities and resources, but also by context factors such as health care structures [11]. For example, in countries with universal health coverage (such as Germany or the UK), individual financial resources are less likely to have a significant impact on treatment burden [12]. Eton et al. [13] further distinguish three components of perceived treatment burden: care-related tasks, strategies for facilitating treatment burden such as seeking support from family or friends, and factors that increase burden, such as problems with medication, financial challenges, or a lack of information. In their *Guideline for Clinical Assessment and Management of Multimorbidity,* the National Institute for Health and Care Excellence recommends monitoring treatment burden and taking action to reduce it where indicated, with the ultimate goal to improve quality of life [14]. In clinical practice, this can be an opportunity to initiate an open dialogue about patients’ preferences and challenges in managing their conditions [15], as well as a first step towards shared-decision making. Similarly, it could help to identify those at risk of being overburdened and resulting non-adherence. However, no instrument is yet available for use in German language.

Several patient-reported instruments for the assessment of treatment burden in patients with multimorbidity have recently been developed for the English-speaking population [16–19]. When assessing these instruments, we found the Multimorbidity Treatment Burden Questionnaire (MTBQ) to be most appropriate for the target population of older adults with multiple chronic conditions and diverse educational backgrounds. Whereas other instruments were longer and more difficult to understand or focused only on specific aspects of treatment burden, the MTBQ stood out for its brevity, intelligibility, and participatory and theoretically informed development [19]. The original MTBQ was tested in a large sample (n = 1,524) of older adults with multimorbidity (defined as the presence of three or more long-term conditions) recruited from general practices in England and Scotland as part of the 3D Study [20]. The MTBQ demonstrated good construct validity, internal consistency and responsiveness. The questionnaire has previously been translated into Danish [5, 21] and Chinese [22]. Its items address aspects of treatment burden that are also relevant to patients navigating the German health care system: Scheduling medical appointments, obtaining prescriptions, challenges related to medication intake, self-management and necessary lifestyle changes, financial aspects and the impact of treatments on personal relationships.

In this study, our objective was to (a) translate and cross-culturally adapt a German version of the MTBQ, (b) validate the adapted version in a sample of older adults with multimorbidity and (c) analyse the relationship between treatment burden scores and sociodemographic characteristics as well as other patient-reported health measures.

## Materials and methods

Following the *Guidelines for Translating and Adapting Tests* by the International Test Commission [23], we used a multiple step translation process and conducted cognitive interviews as well as a pilot test. Psychometric properties of the questionnaire were determined in a sub-study of the MULTIqual project [24], which included a sample of 346 older adults (65 years and older) with three or more long-term conditions.

### Description of the multimorbidity treatment burden questionnaire

The MTBQ is an easy-to-understand questionnaire on the perceived difficulty of health care tasks and their impact on everyday life. The original questionnaire consists of ten items and includes three optional questions (items 3, 9, and 10) that were not applicable in the UK study sample but may be relevant in other populations. The MTBQ was developed based on a literature review and discussions with a patient and public involvement group. In addition, cognitive interviews were conducted to examine content validity. Responses are given on a 5-point Likert scale, with values ranging from 0 (*not difficult* or *does not apply*) to 4 (*extremely difficult*). The global score is computed as the mean score multiplied by 25, resulting in a global score between 0 and 100. The global score can be computed if a person has answered at least 50% of the questions. Four treatment burden groups were categorized in the UK sample: no treatment burden (score 0) and the tertiles low (< 10), medium (10–22) and high treatment burden (≥ 22).

A previous psychometric study of the original MTBQ found positively skewed scores with floor effects for all items. Exploratory factor analysis yielded a one-factor solution with a Cronbach’s Alpha of 0.83 suggesting high internal consistency. Analysis of construct validity revealed a strong positive association with self-reported disease burden (*r*_*s*_ = 0.43, *p* < 0.001), and moderate negative associations with quality of life (*r*_*s*_ = − 0.36, *p* < 0.001) and self-rated health (*r*_*s*_ = − 0.36, *p* < 0.001). Moreover, higher treatment burden scores were significantly associated with a higher number of comorbidities (*r*_*s*_ = − 0.31, *p* < 0.001. Regression analysis showed significant associations of changes in MTBQ scores with changes in measures of health-related quality of life and patient’s assessment of chronic care at nine-month follow-up, suggesting good responsiveness [19].

### Translation

Two translators with in-depth knowledge of the target language, health care system and culture (a researcher with experience in test development and a cultural scientist) carried out the forward translation of the full 13-item version of the MTBQ independently. The two versions were then reconciled into one by both translators. This was followed by two independent backward translations by a psychologist and a professional interpreter. All translators speak German as their mother tongue. The translations were reviewed using the checklist by Hambleton and Zenisky [25] in order to reach consensus on a final version. To ensure cross-cultural validity, ambiguities concerning the different healthcare systems were resolved by consulting the translator group and the author of the original questionnaire.

### Cognitive interviews and pilot test

Applying verbal probing and think-aloud technique, we conducted semi-structured cognitive interviews. The aim was to assess the underlying cognitive mechanisms in item processing according to Tourangeau et al. [26]: comprehension, information retrieval, judgement and response behaviour. At first, patients were asked to verbalize their thoughts when answering the questions. Following this, a series of probe questions was administered to elicit further information on the cognitive process [27]. Interviews were digitally recorded and transcribed verbatim. Analysis of the cognitive protocol was performed question by question to identify difficulties that could potentially lead to responses that did not reflect the intended meaning of the item. The coding corresponded to the categories outlined above. Cognitive interviews were conducted among six patients aged 65 and over with three or more long-term conditions recruited from two GP practices. In addition, we carried out a pilot test in order to determine feasibility and to identify potential problems with test administration. Five researchers piloted the questionnaire in a sample of seven persons living with (multiple) long-term conditions.

### Study setting and data collection

We recruited patients (aged 65 and over) with multimorbidity from GP practices as part of the cross-sectional MULTIqual study on quality of care for older patients with multimorbidity [24, 28]. GP practices in North and South Germany (Hamburg and Heidelberg and surroundings) were randomly selected, stratified by region, and invited to take part in the study. Participating GPs screened their regular practice clientele for the presence of at least three long-term conditions out of a pre-defined list of diagnoses that were found to be associated with high disease burden and lower subjective health status. We excluded patients without sufficient German language skills or ability to give informed consent, patients living in nursing homes and patients in palliative care. Out of 1,243 eligible patients from 35 GP practices, 346 patients agreed to participate (response rate: 27.9%). Following written informed consent, standardized interviews were carried out in their homes or in the GP practice. We collected data on sociodemographic characteristics, health care utilization, course of treatment and medication. Other patient-reported health outcomes were assessed with validated instruments: Medication adherence via MARS-D [29], patient activation via PAM 13-D [30], health-related quality of life and self-rated health via EQ-5D-5L [31], and perceived social support via F-SozU K-14 [32]. Recruitment and data collection took place from April 2019 to March 2020.

### Statistical analysis

We used descriptive statistics in SPSS 25.0 to assess item properties. Assuming a congeneric model with varying means and variances of true values, we calculated McDonald’s omega as reliability coefficient using the *MBESS* package for R [33], with scores above 0.70 considered sufficient given the length and purpose of the instrument [34]. We conducted an exploratory factor analysis to assess the dimensionality of the questionnaire. Underlying factors were extracted according results of scree plot, parallel analysis of eigenvalues and Velicer’s minimum average partial test with the R package *EFA.dimensions* [35]. Confirmatory factor analysis was computed using R package *lavaan* [36]. Goodness of fit was examined using the following indices: Robust root mean square error of approximation (RMSEA) with values below 0.05 indicating good model fit and values between 0.05 and 0.08 indicating acceptable model fit; standardized root mean square residual (SRMR) with values below 0.05 indicating good model fit and values between 0.05 and 0.10 indicating acceptable model fit; and comparative fit index (CFI) with values below 0.90 indicating poor model fit [37].

In examining construct validity, we used bivariate correlation analyses to assess the relationship between MTBQ scores and related measures. We hypothesized a positive correlation with number of long-term conditions and negative correlations with medication adherence, patient activation, health-related quality of life and self-rated health, as previously established for several self-report treatment burden questionnaires [6, 18, 19, 38]. Furthermore, we expected lower levels of social support to be associated with greater treatment burden. Unfortunately, there was no comparator scale available to assess concurrent validity. Mann–Whitney U tests were used to analyse the associations between participant characteristics and treatment burden scores. We expected higher treatment burden scores for female respondents and respondents with mental health conditions, as similar results were previously reported for the UK version [19].

## Results

### Translation, cognitive interviews and pilot test

Discrepancies between forward and backward translation as well as the final review highlighted problems in the adaptation of optional item 10: *“Getting help from community services”*. Due to the different organization of the health care systems, there is no analogous service structure in Germany. Although an item was created to determine treatment burden pertaining to services such as physiotherapy and mobile nursing service, cognitive interviews revealed that respondents already included these services under item 6: *“Arranging appointments with health professionals”*. Accordingly, the adaptation led to ambiguities and queries and therefore, we decided to exclude item 10. All other items were found to be applicable to the German health care system.

Furthermore, respondents reported that it was difficult to distinguish between the response categories *“does not apply”* and *“not difficult”*, as the German translations are commonly used for similar purposes. For this reason, we added an instruction to further distinguish between both options*: “It is also possible that some of the aspects do not play a role in your care and do not apply to your situation”*. Cognitive interviews showed that this addition was sufficient to enable a clearer distinction between the two responses. Interviewed patients listed a wide range of health care providers for answering questions 6 to 8, indicating a comprehensive understanding of these items. Likewise, a number of monitoring behaviours were specified for item 5. Respondents did not describe any further aspects of treatment burden not covered by the questionnaire, so that we assume no limitations to the face validity of the German adaptation. The results of the cognitive interviews are presented in more detail in Additional file 1. In the pilot test, it took about 4 min to answer the questionnaire, indicating little burden for respondents. There were no suggestions for any further changes.

### Statistical analysis

Out of the initial sample of 346 patients who participated in the main study, 344 patients responded to at least half of the MTBQ questions, which allowed inclusion in the statistical analysis. Table 1 illustrates the characteristics of the participants. The mean age was 77.5 years with a range from 65 to 97. The sample included slightly more female (55.2%) than male participants. The majority (56.4%) had less than ten years of school education which equates to CASMIN (Comparative Analysis of Social Mobility in Industrial Nations classification of education) grade 1 [39]. On average, participants reported 9.8 long-term conditions.

**Table 1:** Characteristics of the study sample

Characteristics	Patient sample (*N* = 344)
Age: mean ± SD	77.5 ± 7.1
Gender	
Female	190 (55.2%)
Male	154 (44.8%)
Marital status	
Married	189 (54.9%)
Widowed	99 (28.8%)
Divorced	33 (9.6%)
Single	23 (6.7%)
Education level (via CASMIN [39])	
Primary	193 (56.4%)
Secondary	95 (27.8%)
Tertiary	54 (15.8%)
Country of birth	
Germany	319 (92.7%)
Other	25 (7.3%)
Nursing care dependency	
Yes	78 (22.7%)
No	266 (77.3%)
Total number of comorbidities: mean ± SD	9.8 ± 4.5
Total number of long-term medications: mean ± SD	6.0 ± 3.2
Comorbidities	
Chronic low back pain	196 (57.0%)
Urinary incontinence	136 (39.5%)
Diabetes	119 (34.6%)
Coronary heart disease	71 (20.6%)
COPD	71 (20.6%)
Heart failure	61 (17.7%)
Depression	51 (14.8%)
Asthma	50 (14.5%)
Chronic kidney disease	41 (11.9%)
Stroke/Transient ischaemic attack	31 (9.0%)
Anxiety disorder	27 (7.8%)
Parkinson’s disease	5 (1.5%)
Treatment burden level (as assessed via MTBQ global score)	
No burden (0)	88 (25.6%)
Low burden (< 10)	134 (39.0%)
Medium burden (11–22)	97 (28.2%)
High burden (≥ 22)	25 (7.3%)

#### Item properties

Proportion of missing data was less than 1% for each item (see Table 2). We observed floor effects for all items, with positively skewed distributions (Skewness = 2.02, Kurtosis = 4.72). Following the instruction of the original questionnaire, all items with more than 40% *“does not apply”* responses were excluded from the statistical analysis, which led to the removal of item 9. However, in contrast to the original questionnaire, we retained item 3, as this applied to most respondents. The mean score was multiplied by 25 to obtain the global MTBQ score. Descriptive statistics are provided in Table 3. For the purpose of comparison, we retained the original thresholds for categorizing the global score into four levels of treatment burden as applied to the UK, Chinese, and Danish versions. As a result, 25.6% of the study population showed no treatment burden, 39.0% low treatment burden, 28.2% medium treatment burden, and 7.3% high treatment burden (see Table 1). Mann–Whitney U tests showed that global MTBQ scores were significantly higher in female (*Mdn*_1_ = 6.82) than in male participants (*Mdn*_2_ = 4.55; *U* = 11,729, *p* = 0.001) and higher for persons with anxiety or depression (*Mdn*_1_ = 9.10, *Mdn*_2_ = 4.55; *U* = 3172, *p* = 0.024).

**Table 2:** Distribution of responses (*N* = 344)

Items	*n*	Not difficult (in %)	A little difficult (in %)	Quite difficult (in %)	Very difficult (in %)	Extremely difficult (in %)	Does not apply (in %)
1. Taking lots of medications	342	65.2	10.2	5.8	2.6	2.3	13.7
2. Remembering how and when to take medication	344	84.3	8.7	0.9	0.0	0.9	5.2
3. Paying for prescriptions, over the counter medication or equipment	342	68.1	14.3	5.0	1.8	0.9	9.9
4. Collecting prescription medication	344	78.8	4.4	1.2	0.6	2.3	12.8
5. Monitoring your medical conditions (eg, checking your blood pressure or blood sugar, monitoring your symptoms, etc.)	344	84.0	4.1	1.7	0.6	0.3	9.3
6. Arranging appointments with health professionals	344	82.0	7.3	2.9	1.5	1.5	4.9
7. Seeing lots of different health professionals	343	69.1	12.2	6.1	4.4	1.7	6.4
8. Attending appointments with health professionals (eg, getting time off work, arranging transport, etc.)	344	76.2	4.1	2.9	2.9	1.5	12.5
9. Getting health care in the evenings and at weekends	344	36.6	4.7	2.6	1.7	1.2	53.2
11. Obtaining clear and up-to-date information about your condition	341	84.5	5.6	2.9	1.2	0.6	5.3
12. Making recommended lifestyle changes (eg, diet and exercise)	343	52.8	26.5	8.7	4.4	0.6	7.0
13. Having to rely on help from family and friends	342	36.8	16.7	8.8	6.4	2.0	29.2

Item 9 was excluded from the analysis, as it was not applicable for most participants. This item may be relevant to different populations and is therefore retained as an optional question for use in other appropriate settings. (Optional) item 10 of the English version was excluded from the German MTBQ due to lack of transferability to the German health care system

**Table 3:** Descriptive statistics of health measures and correlation to global MTBQ scores

	*N*	Mean	Range	Std. Deviation	Spearman rank correlation to global MTBQ score (*r*_*s*_)	*p*
German MTBQ: Treatment burden	344	7.81	0.00–52.27	9.46	–	–
PAM-13: Patient activation	341	76.10	22.60–100.00	16.43	− 0.457	< 0.001
MARS-D: Medication adherence	335	24.01	8.00–25.00	1.87	− 0.222	< 0.001
F-SozU K14: Perceived social support	327	4.36	1.57–5.00	0.67	− 0.308	< 0.001
EQ-5D-5L utility score: Health-related QoL	336	0.75	− 0.14–1.00	0.23	− 0.351	< 0.001
EQ VAS: Self-rated health	338	63.05	0–100	20.65	− 0.335	< 0.001

*QoL* quality of life, *VAS* visual analogue scale

#### Dimensionality and reliability

The Kaiser–Meyer–Olkin (KMO) measure of sampling adequacy (0.755) indicated that the data was suitable for exploratory factor analysis. Although Kaiser Guttman criterion determined a four-factor solution, the scree plot (see Additional file 2) and both parallel analysis of eigenvalues and Velicer’s Minimum Average Partial test (MAP) suggested one common factor, which explained 21.20% of total variance. Model indices for the confirmatory factor analysis showed acceptable to slightly less than good model fit: χ^2^ = 86.917 (44), *p* = 0.000, Robust CFI = 0.845, RMSEA = 0.073, SRMR = 0.072. Internal reliability was satisfactory with *ω*_*t*_ = 0.71.

#### Construct validity

We found a positive association between the global MTBQ score and patient-reported number of comorbidities (*r*_*s*_ = 0.409, *p* < 0.001). There was an association between greater burden and lower health-related quality of life (*r*_*s*_ = − 0.351, *p* < 0.001) and self-rated health (*r*_*s*_ = − 0.335, *p* < 0.001). We found negative associations for social support (*r*_*s*_ = − 0.308, *p* < 0.001), patient activation (*r*_*s*_ = − 0.457, *p* < 0.001) and medication adherence (*r*_*s*_ = − 0.222, *p* < 0.001) as well. Our results (see Table 3) support all our hypotheses on construct validity, with coefficients comparable to the results shown for the UK version of the MTBQ.

## Discussion

In this study, we developed a German tool to measure treatment burden in patients with multimorbidity. We used a thorough and well-established methodology to translate and adapt a German version of the MTBQ. The questionnaire contains 11 core items and one optional item that was not applicable to our study population but may be relevant for other settings. Cognitive interviewing and piloting led to smaller adaptations and demonstrated overall good content validity. Statistical analysis was performed within a cross-sectional design. Validity analysis yielded significant associations to related constructs as hypothesized, similar to the results reported for other instruments assessing treatment burden [19, 40]. While factor analysis indicated a single-factor solution, the proportion of explained variance and model fit were only satisfactory. The UK version showed one-dimensionality, whereas other psychometric studies suggested alternative three-factor solutions for the Chinese and Danish versions [21, 22]. Due to these differences, we assume that the underlying construct is a formative model rather than a reflective model and therefore dimensionality is less relevant for this instrument [41]. The difference between formative and reflective models in psychometric validation mainly relates to the internal structure of the patient-reported outcome measure. In a reflective model, one latent variable manifests itself in all indicators, i.e., in the items of a (sub-) scale, so that the items are expected to be positively correlated. In a formative model, on the other hand, the measured items constitute the latent variable, with no presumption of inter-item correlation [42]. Regarding the construct of treatment burden, our empirical results support theoretical assumptions: The manifestation of treatment burden is influenced by relatively stable individual factors such as diseases, resources and coping strategies, while it varies as a function of the workload resulting from treatment regimens and also the organisational requirements of health care systems. While this finding suggests limited utility of factor analysis, it also underscores the importance of cross-cultural adaptation and qualitative pretesting. The distinct floor effects of the scale are similarly reported for other instruments on treatment burden [16, 43], in some cases even to a greater extent [18].Nevertheless, in patients with low burden, the responsiveness to improvement may be limited.

In our sample, patients scored lower on average for treatment burden than in the UK sample of Duncan et al. [19]. These differences could be explained by the dense medical care structure in the regions of our study centres and the older age of our participants. Previous studies found that treatment burden tends to be greater in younger patients [19, 40, 44]. One possible explanation may be that younger people not only experience stronger life demands and requirements regarding to their social roles but also have different expectations of their general functioning. The longer patients live with long-term conditions, the more likely they are to adjust to everyday treatment requirements, such as taking medication regularly or monitoring symptoms.

We were surprised to see that item 3 on financial burden was relevant to most of our participants, in contrast to the original UK sample. In outpatient care in Germany, out-of-pocket costs of statutory health insurance only apply to co-payments for medication, supportive therapies and medical aids. For patients with long-term conditions, there is a burden limit of 1% of their income. Even so, not all patients use this exemption or are even aware of this possibility. On average, the highest scores were obtained for item 12 (*“Making recommended lifestyle changes”*) and item 13 (*“Having to rely on help from family and friends”*). These findings corroborate the relevance of intervention studies to target these aspects of care.

One major advantage in our study is that the sample represents the most prominent target group of multimorbidity research. On the other hand, the sample restrictions might pose a limitation to the generalizability of these findings to younger patients and patients with fewer comorbidities. Moreover, it should be noted that our data collection is confronted with a possible self-selection bias: those who already perceive a greater burden and feel overwhelmed might be less likely to respond to a study invitation. Due to the cross-sectional design, we were not able to establish the predictive value of the German MTBQ and to explore the possible effect of overburden on non-adherence. One psychometric property we did not examine was test–retest reliability, which should be addressed in the future. Despite our promising results, additional research is required to determine the responsiveness of the German MTBQ in longitudinal designs and the clinical utility of this tool in primary care settings. Future studies will be needed to confirm and advance the threshold values for categorizing MTBQ scores into four levels of treatment burden, ideally based on a clinical anchor [21]. Further work is required to shed light on the impact of life demands, social roles, coping behaviour and resources on treatment burden to allow a better understanding of our findings.

## Conclusions

The German MTBQ is a brief and concise patient reported outcome measure to examine perceived treatment burden in patients with multimorbidity. It demonstrated good psychometric properties and is the first valid instrument to assess treatment burden in patients with multiple long-term conditions in Germany. Since cross-culturally adapted and validated versions of the MTBQ are available in several languages, it could also be used in international research studies. With its short administration time, the MTBQ may be suitable to detect people experiencing high burden in clinical practice, although its use in as a clinical tool has not yet been validated. Implementing the MTBQ as a clinical tool has the potential to inform shared decision-making and elicit areas where patients need additional support.

## Supplementary Information

**Additional file 1.** Results of cognitive interviews.

**Additional file 2** Exploratory factor analysis: Scree plot for German MTBQ.

## Data Availability

The analysed dataset is available from the corresponding author on reasonable request. The MTBQ can be requested from: https://www.bristol.ac.uk/primaryhealthcare/resources/mtbq/

## References

[1] Koné Pefoyo AJ, Bronskill SE, Gruneir A, Calzavara A, Thavorn K, Petrosyan Y (2015). The increasing burden and complexity of multimorbidity. BMC Public Health.

[2] Kingston A, Robinson L, Booth H, Knapp M, Jagger C (2018). Projections of multi-morbidity in the older population in England to 2035: estimates from the population ageing and care simulation (PACSim) model. Age Ageing..

[3] Lesage A, Leclère B, Moret L, Le Glatin C (2021). Decreasing patient-reported burden of treatment: a systematic review of quantitative interventional studies. PLoS ONE.

[4] Gallacher KI, May CR, Langhorne P, Mair FS (2018). A conceptual model of treatment burden and patient capacity in stroke. BMC Fam Pract.

[5] Friis K, Lasgaard M, Pedersen MH, Duncan P, Maindal HT (2019). Health literacy, multimorbidity, and patient-perceived treatment burden in individuals with cardiovascular disease. A Danish population-based study. Patient Educ Couns..

[6] Tran V-T, Harrington M, Montori VM, Barnes C, Wicks P, Ravaud P (2014). Adaptation and validation of the treatment burden questionnaire (TBQ) in English using an internet platform. BMC Med.

[7] Tran V-T, Montori VM, Ravaud P (2020). Is my patient Overwhelmed?: determining thresholds for acceptable burden of treatment using data from the compare e-Cohort. Mayo Clin Proc.

[8] Jonas DE, Ibuka Y, Russell LB (2011). How much time do adults spend on health-related self-care? results from the american time use survey. J American Board Fam Med.

[9] Boyd CM, Darer J, Boult C, Fried LP, Boult L, Wu AW (2005). Clinical practice guidelines and quality of care for older patients with multiple comorbid diseases: implications for pay for performance. JAMA.

[10] Shippee ND, Shah ND, May CR, Mair FS, Montori VM (2012). Cumulative complexity: a functional, patient-centered model of patient complexity can improve research and practice. J Clin Epidemiol.

[11] Rosbach M, Andersen JS (2017). Patient-experienced burden of treatment in patients with multimorbidity - a systematic review of qualitative data. PLoS ONE.

[12] Abu Dabrh AM, Gallacher K, Boehmer KR, Hargraves IG, Mair FS (2015). Minimally disruptive medicine: the evidence and conceptual progress supporting a new era of healthcare. J R Coll Physicians Edinb.

[13] Eton DT, de Oliveira DR, Egginton JS, Ridgeway JL, Odell L, May CR, Montori VM (2012). Building a measurement framework of burden of treatment in complex patients with chronic conditions: a qualitative study. Patient Relat Outcome Meas..

[14] National Institute for Health and Care Excellence (NICE). Multimorbidity: clinical assessment and management. 2016. https://www.nice.org.uk/guidance/ng56. Accessed 9 Dec 2020.

[15] Ridgeway JL, Egginton JS, Tiedje K, Linzer M, Boehm D, Poplau S (2014). Factors that lessen the burden of treatment in complex patients with chronic conditions: a qualitative study. Patient Prefer Adherence.

[16] Tran V-T, Montori VM, Eton DT, Baruch D, Falissard B, Ravaud P (2012). Development and description of measurement properties of an instrument to assess treatment burden among patients with multiple chronic conditions. BMC Med.

[17] Eton DT, Yost KJ, Lai J-S, Ridgeway JL, Egginton JS, Rosedahl JK (2017). Development and validation of the patient experience with treatment and self-management (PETS): a patient-reported measure of treatment burden. Qual Life Res.

[18] Boyd CM, Wolff JL, Giovannetti E, Reider L, Weiss C, Xue Q (2014). Healthcare task difficulty among older adults with multimorbidity. Med Care.

[19] Duncan P, Murphy M, Man M-S, Chaplin K, Gaunt D, Salisbury C (2018). Development and validation of the multimorbidity treatment burden questionnaire (MTBQ). BMJ Open.

[20] Salisbury C, Man M-S, Bower P, Guthrie B, Chaplin K, Gaunt DM (2018). Management of multimorbidity using a patient-centred care model: a pragmatic cluster-randomised trial of the 3D approach. The Lancet.

[21] Pedersen MH, Duncan P, Lasgaard M, Friis K, Salisbury C, Breinholt LF (2022). Danish validation of the multimorbidity treatment burden questionnaire (MTBQ) and findings from a population health survey: a mixed-methods study. BMJ Open.

[22] Dou L, Huang J, Duncan P, Guo L (2020). Translation, cultural adaptation and validation of the Chinese multimorbidity treatment burden questionnaire (C-MTBQ): a study of older hospital patients. Health Qual Life Outcomes.

[23] International Test Commission. The ITC Guidelines for Translating and Adapting Tests. 2017. www.InTestCom.org. Accessed 21 Apr 2021.

[24] Schulze J, Glassen K, Pohontsch NJ, Blozik E, Eißing T, Breckner A (2022). Measuring the quality of care for older adults with multimorbidity: results of the MULTIqual project. Gerontologist.

[25] Hambleton RK, Zenisky AL, Matsumoto D, van de Vijver FJR (2010). Translating and adapting tests for cross-cultural assessments. Cross-cultural research methods in psychology.

[26] Tourangeau R, Rips LJ, Rasinski K (2000). The psychology of survey response.

[27] Pohontsch N, Meyer T (2015). Das kognitive Interview-Ein Instrument zur Entwicklung und Validierung von Erhebungsinstrumenten. [Cognitive Interviewing–A Tool to Develop and Validate Questionnaires]. Rehabilitation (Stuttg).

[28] Pohontsch NJ, Schulze J, Hoeflich C, Glassen K, Breckner A, Szecsenyi J (2021). Quality of care for people with multimorbidity: a focus group study with patients and their relatives. BMJ Open.

[29] Mahler C, Hermann K, Horne R, Ludt S, Haefeli WE, Szecsenyi J, Jank S (2010). Assessing reported adherence to pharmacological treatment recommendations. Translation and evaluation of the medication adherence report scale (MARS) in Germany. J Eval Clin Pract..

[30] Brenk-Franz K, Hibbard JH, Herrmann WJ, Freund T, Szecsenyi J, Djalali S (2013). Validation of the German version of the patient activation measure 13 (PAM13-D) in an international multicentre study of primary care patients. PLoS ONE.

[31] The EuroQol Group (1990). EuroQol-a new facility for the measurement of health-related quality of life. Health Policy.

[32] Fydrich T, Sommer G, Tydecks S, Brähler E (2009). Fragebogen zur sozialen Unterstützung (F-SozU): Normierung der Kurzform (K-14) [Social Support Questionnaire (F-SozU): standardization of short form (K-14).]. Z Med Psychol.

[33] Kelley K (2007). Methods for the behavioral, educational, and social sciences: an R package. Behav Res Methods.

[34] Nunnally JC (1978). Psychometric theory.

[35] O'Connor BP. Exploratory Factor Analysis Functions for Assessing Dimensionality. 2021. https://cran.r-project.org/web/packages/EFA.dimensions/EFA.dimensions.pdf. Accessed 22 Apr 2021.

[36] Rosseel Y (2012). lavaan: an R package for structural equation modeling. J Stat Soft.

[37] Kline RB (2016). Principles and practice of structural equation modeling.

[38] Eton DT, Linzer M, Boehm DH, Vanderboom CE, Rogers EA, Frost MH (2020). Deriving and validating a brief measure of treatment burden to assess person-centered healthcare quality in primary care: a multi-method study. BMC Fam Pract.

[39] Brauns H, Scherer S, Steinmann S, Hoffmeyer-Zlotnik JHP, Wolf C (2003). The CASMIN educational classification in international comparative research. Advances in cross-national comparison: a European working book for demographic and socio-economic variables.

[40] Spencer-Bonilla G, Quiñones AR, Montori VM (2017). Assessing the burden of treatment. J Gen Intern Med.

[41] Mokkink LB, Prinsen CA, Patrick DL, Alonso J, Bouter LM, Vet HC de, Terwee CB. COSMIN methodology for systematic reviews of Patient‐Reported Outcome Measures (PROMs): user manual. 2017. https://www.cosmin.nl/wp-content/uploads/COSMIN_manual_syst-review-PROMs_V1.0.pdf. Accessed 5 May 2021.

[42] Coltman T, Devinney TM, Midgley DF, Venaik S (2008). Formative versus reflective measurement models: two applications of formative measurement. J Bus Res.

[43] Gibbons CJ, Kenning C, Coventry PA, Bee P, Bundy C, Fisher L, Bower P (2013). Development of a multimorbidity illness perceptions scale (MULTIPleS). PLoS ONE.

[44] Herzig L, Zeller A, Pasquier J, Streit S, Neuner-Jehle S, Excoffier S, Haller DM (2019). Factors associated with patients’ and GPs’ assessment of the burden of treatment in multimorbid patients: a cross-sectional study in primary care. BMC Fam Pract.

