# Supplementary material for: Adaptation and validation of a German version of the Multimorbidity Treatment Burden Questionnaire
Source: Health Qual Life Outcomes. 2022 Jun 3;20:90. doi: 10.1186/s12955-022-01993-z (PMC9166496; doi:10.1186/s12955-022-01993-z)
Supplement: Supplementary file 1 — Additional file 1. Results of cognitive interviews. [file 12955_2022_1993_MOESM1_ESM.pdf]

Supplement to “Adaptation and validation of a German version of the Multimorbidity Treatment Burden Questionnaire”

Additional file 1

Table 1 Results of cognitive interviews (in excerpts)

**Citation**	**Cognitive process*** ***(Comprehension, information retrieval, judgement and response behaviour)***	**Evaluation**	**Required revisions**
**Item 1: Taking lots of medications**			
I: “How did you come up with your answer?”<br>P2: “Because I can’t swallow my pills.” (…)<br>I: “So for you it is more about the difficulty of swallowing your medication?”<br>P2: “Yes, well, I always need at least half a glass to able to swallow them.”	Judgement	Understood intended meaning	None
P4: “Not anymore. I've gotten used to it. (...) No, the idea of taking these little pieces here. I don't know exactly what's in them. The thought ‘what are they all going to do now?’ was difficult for me at first. But after a while it was just business as usual.”	Judgement	Takes changes over time into account	None
P6: “Not at all. (...) I have, I have always done well. And that is, do you want to see a box like that? (...) So, they're placed in here for morning, noon, and night, and I don't need any at noon, just morning and night. And then it's six at a time and I put them in here like this, do that and drink properly afterwards.”	Information retrieval	Recounts strategies associated with the management of medication intake	None
**Item 2: Remembering how and when to take medication**			
P6: “No, it's not difficult for me. Between half past eight and ten in the morning and in the evening I take it half past four. That way I don't have to run to the bathroom at night.”	Information retrieval	Recounts behavior associated with the management of medication intake	None

**Item 3: Paying for prescriptions, over the counter medication or equipment**			
P3: “That's not difficult for me either, because then I call there and then I have this, what's it called, this…”<br>I: “…this exemption”<br>P3: “This exemption and then I make a pre-payment once a year to the health insurance company and then everything is fine.”	Judgement	Refers to strategies to minimize financial burden	None
P2: “For me it's hard (...) And if you only have a small pension (...) Then it's pretty tough already.”<br>I: “And from the response categories, what do you come up with here?”<br>P2: “Yeah, it's quite difficult for me (...)”	Judgement	Takes own financial hardship into account	None
**Item 4: Collecting prescription medication**			
P3: „What do you mean now? (...) Picking up prescriptions or picking them up from the pharmacy? (...) If I am in the practice, then of course I get it and if I am not, then either the practice sends it to me or my son picks up the prescription. (...) And then I send the prescription to a pharmacy, and they send it to me.”<br>I: “Ah okay, so there you have this modus, so to speak.”<br>P3: “Exactly.”<br>I: “And then, do you find that difficult?”	Comprehension	Initial confusion about intended meaning, but no misunderstanding	None
P3: “No, I only need to put it in the mailbox or my son puts the envelope in the mailbox or my daughter puts it in the mailbox. So I have practically nothing to do with it. I have a prepaid envelope from the pharmacy and then (...) yes, it works.”	Information retrieval	All necessary actions to collect prescription medication are being retrieved	None
**Item 5: Monitoring your medical conditions (eg, checking your blood pressure or blood sugar, monitoring your symptoms, etc)**			
P5: “Yes, I have to say, quite difficult.”<br>I: “How do you arrive at your answer, what do you find so difficult, that is, quite difficult?”<br>P5: “I find it sometimes annoying that you must think about it.”	Response behaviour	Differentiated response using the full range of the scale	None

P6: “Not difficult. I have a blood pressure monitor myself, and besides, it's very well calibrated.”	Judgement	Information on self-monitoring is retrieved comprehensively	None
**Item 6: Arranging appointments with health professionals**			
I: “Who would fall under this category for you?<br>P1: “The appointments?”<br>I: “Yes.”<br>P1: “Orthopaedist, eye doctor (...) and cardiology, of course. Dermatologist, if necessary, something like that.”<br>I: “What about physical therapy?”<br>P1: “And them. (...) But I usually do it online all the time.”	Comprehension	Understood intended meaning, a number of physicians and other health professionals are listed who were referred to when answering the question	None
**Item 7: Seeing lots of different health professionals**			
P6: “There are not so many. (...) That is hospital X, the GP, dentist. No, that's not much and they are all close to us.”	Comprehension	Takes all involved health professionals into account	None
**Item 8: Attending appointments with health professionals (eg, getting time off work, arranging transport, etc)**			
P3: “That works wonderfully, since I have this exemption, I have a hundred percent severe disability from the fund and walking disability and so I go everywhere by cab. (...) The health insurance covers that.”	Judgement	Takes benefits into account when judging difficulty	None
P2: “No, that does not apply.” (…)<br>I: “And which health professionals did you think of that you have to go to?”<br>P2: “The one who is responsible for my tendons. This is a specialist in city X and, yes, and my ophthalmologist.” (…)<br>I: “So that' s not a difficulty for you?”<br>P2: “We do a lot of things together, because my husband also has to go to the doctor and so on, and then we always do it in a way	Comprehension<br><br><br><br><br>Judgement	Problematic - difficulty in differentiating between “does not apply” and “not difficult”<br><br>Refers to actions necessary to attend medical appointments	Addition of an explanation of the category “does not apply” to the instructions<br><br>None

that we schedule it for one day and at one time, so that you don't have to go there twice.”			
**Item 9: Getting health care in the evenings and at weekends**			
P2: “So you mean when the medication runs out or something? (...) We check in advance, or when the doctors go on vacation or so, we check in advance whether we have enough pills and other things.”	Comprehension	Irritated by question, as apparently not applicable	Addition of an explanation of the category “does not apply” to the instructions
P1: “Yes, there is now this ‘116-117’ number that you can call [for medical assistance out-of-hours]. I did that once, where they told me, ‘You absolutely have to get the emergency doctor’. (...) That was quite difficult for me. (...) When I was with my parents and when there was a problem, I had no difficulty with that at all. I reacted immediately and switched. But for myself, I'm not always good at it.”	Judgment	Takes own attitude towards getting help from emergency care into account	None
**Item 10: Getting help from community services (e.g. physiotherapy, district nurses etc.)**			
P3: “How do you mean, community services? (…) The home assistance comes here once a week and mops and vacuums. (…) I go [to physical therapy] by cab. And there is a lift and so I get there.”	Comprehension	Problematic - the content of the question is not immediately clear to the respondent	Removal of question as it is not understood by respondents due to lack of transferability to German health care system
P6: “No, as I said, my doctor does not make home visit. (…) But an emergency doctor will always come to me. So, no, not difficult.”	Comprehension	Problematic - when answering the question, other content is referred to than intended with the item	Removal of question as it is not understood by respondents due to lack of transferability to

			German health care system
**Item 11: Obtaining clear and up-to-date information about your condition**			
P4: “Well, I’m not yet that good with Google, because I haven’t done it that long. But on the television, from time to time I watch a program on nutrition, but I don’t look up on the internet ‘What is hypertension’ – I mean, I already know that.”	Judgement	Refers to behaviour they engage in to obtain information	None
P5: “A little difficult. (…) Well, if I want to know something, I ask. And then I get the information from the doctors. So a little difficult is quite right. You have to ask.“	Response behaviour	Uses the small-step scale for differentiation	None
**Item 12: Making recommended lifestyle changes (e.g. diet and exercise etc.)**			
P3: “Yes, as I said, I already told you that I exercise, which of course is insanely difficult for me, the exercise. But I do it because you need oxygen for the blood circulation and everything. And I've actually always eaten sensibly, so not a lot of meat and so on, but lots of salad, vegetables, potatoes and so twice a week at most meat or fish.”<br>I: “So you already follow a healthy diet.”<br>P3: “I already do that, I always have, practically from my childhood.”<br>I: “While it is different with exercising?”<br>P3: “Exercise is hard, yes, because I can't move anymore. I used to walk a few kilometres a day or ride a bike and of course it’s not like that anymore. (...) And that is also incredibly exhausting. (…)”<br>I: “And what would you say, how difficult it is all in all for you?”<br>P3: “Actually, extremely difficult.”	Information retrieval<br><br><br><br><br><br><br><br><br><br>Response behaviour	Refers comprehensively to lifestyle changes<br><br><br><br><br><br><br><br><br><br>Differentiated response using the full range of the scale	None<br><br><br><br><br><br><br><br><br><br>None
I: “Exactly why did you choose ‘does not apply’?”<br>P2: “Yes, not difficult. Does not apply.”	Response behaviour	Problematic - difficulty in differentiating between	Addition of an explanation of the category “does not

I: “So would you say that doesn't apply to you at all, that you have to change your lifestyle?”<br>P2: “Well, I, we changed it, in that we don't eat so much meat anymore and all these things, but, no, that's actually normal for me.”		“does not apply” and “not difficult”	apply” to the instructions
**Item 13: Having to rely on help from family and friends**			
P3: “Very difficult. (...) Yes, it's a strange feeling when you've been able to do everything and then you sit there and say, well, fine. They all have, my daughter, my son, they all have a job and that is of course a rather underwhelming feeling.”	Judgement	Takes own attitudes towards getting help into account	None
P1: “Difficult. I don't like to do that. (...) I always prefer to try everything myself first.”<br>I: “Yes. And on this scale, what would you say? Please think aloud.”<br>P1: “Yes. Quite difficult.”<br>I: “So that is an issue...”<br>P1: “Yes because I am used to it. Most of my life I lived alone (...) When my father was still alive, that was of course great. He always helped me a lot and did technical things like that.”	Judgement	Takes own attitudes towards getting help into account	None

Notes: * Based on cognitive mechanisms in item processing by Tourangeau et al. [26]

Main results

Overall good comprehension of intended meaning of items

Response scale allows for broad differentiation

Items comprehensively capture the content of the latent construct, no additions resulting from follow-up questions

Required revisions:

- Addition of an explanation of the category “does not apply” to the instructions
- Item 10: Removal of question as it is not understood by respondents due to lack of transferability to German health care system
