# Supplementary figures and images for: Adaptation and validation of a German version of the Multimorbidity Treatment Burden Questionnaire
Source: Health Qual Life Outcomes. 2022 Jun 3;20:90. doi: 10.1186/s12955-022-01993-z (PMC9166496; doi:10.1186/s12955-022-01993-z)

**Additional file 2**

**Figure 1** Scree plot for German MTBQ

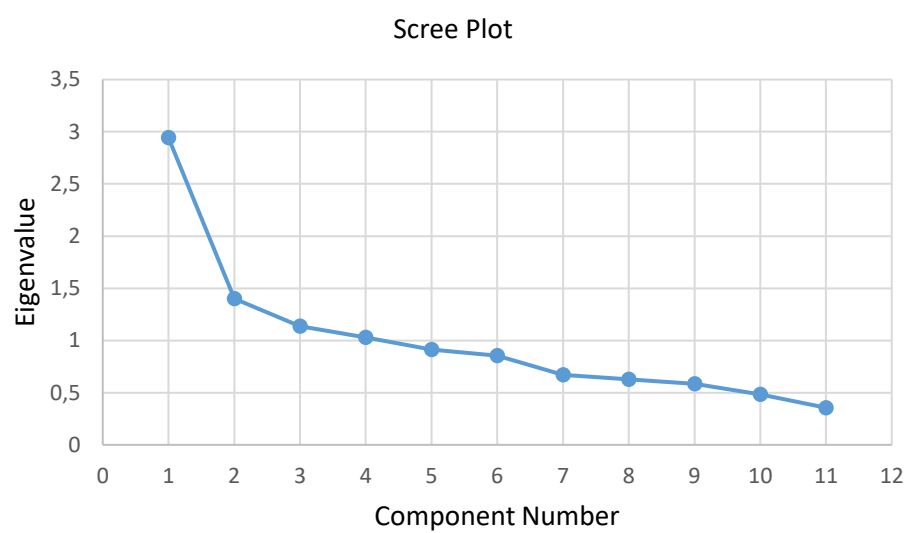

Supplement: Supplementary file 2 — Additional file 2 Exploratory factor analysis: Scree plot for German MTBQ. [file 12955_2022_1993_MOESM2_ESM.pdf]
